# Navigating Complexity: A Rare Case of Down Syndrome With Dural Venous Thrombosis in a Neonate

**DOI:** 10.7759/cureus.52982

**Published:** 2024-01-26

**Authors:** SreeHarsha Damam, Revat J Meshram, Amar Taksande, Sagar Karotkar, Mahaveer S Lakra, Aditi Rawat, Astha Khurana, Chaitanya Kumar Javvaji, Naramreddy Sudheesh Reddy, Sri Sita Naga Sai Priya K

**Affiliations:** 1 Pediatrics, Jawaharlal Nehru Medical College, Datta Meghe Institute of Higher Education and Research, Wardha, IND; 2 Neonatalogy, Jawaharlal Nehru Medical College, Datta Meghe Institute of Higher Education and Research, Wardha, IND

**Keywords:** neurophysiotherapy, aspiration pneumonia, respiratory distress, multidisciplinary management, neonate, dural venous thrombosis, down syndrome

## Abstract

This case report presents a unique clinical scenario of a 2 kg male neonate with Down syndrome complicated by dural venous thrombosis. Born via normal vaginal delivery, the infant exhibited syndromic features characteristic of Down syndrome, necessitating admission to the neonatal intensive care unit (NICU) for respiratory distress. Confirmatory karyotyping established the diagnosis. Subsequent complications included germinal matrix haemorrhage, hypoxic-ischemic encephalopathy, and aspiration pneumonia. An MRI revealed dural venous thrombosis in the left transverse sinus, an uncommon manifestation in neonates with Down syndrome. Multidisciplinary management involved respiratory support, antibiotic therapy, and neurophysiotherapy. Infectious complications, including *Klebsiella pneumoniae* growth, required tailored antibiotic intervention. Despite intubation and CO_2_ retention challenges, the neonate improved and was ultimately discharged with favourable anthropometric measurements. This case underscores the importance of a comprehensive approach to neonatal care in the context of Down syndrome, emphasising the need for early recognition and management of rare complications such as venous thrombosis. The positive outcome highlights the efficacy of a multidisciplinary strategy in addressing complex neonatal conditions.

## Introduction

Down syndrome, also known as Trisomy 21, is a chromosomal disorder characterized by the presence of an extra copy of chromosome 21. This genetic anomaly leads to a range of physical and developmental features, making neonates with Down syndrome susceptible to various health complications [1]. Common manifestations include syndromic facial features, hypotonia, and congenital heart defects, necessitating specialized care and early intervention [2]. Respiratory distress is a frequent concern in neonates with Down syndrome due to anatomical and functional abnormalities in the upper airway and respiratory system [3]. The risk of congenital heart defects also contributes to the complexity of managing these infants in the neonatal period [4]. While the literature extensively documents these aspects, there is limited information on the association between Down syndrome and venous thrombosis in the neonatal population.

Venous thrombosis in neonates is an uncommon but potentially severe condition that poses diagnostic and therapeutic challenges. The neonatal coagulation system, influenced by various factors, differs significantly from that of older children and adults [5]. Thrombotic events in neonates are often associated with underlying medical conditions, central venous catheters, or infections [6]. However, the specific association of venous thrombosis with Down syndrome in neonates is rarely reported.

This case report contributes to the literature by presenting a rare occurrence of dural venous thrombosis in a neonate with Down syndrome. The aim is to highlight the need for heightened awareness among healthcare providers regarding the potential complications in this population, including thrombotic events. Understanding the multifaceted nature of Down syndrome and its associated complications is essential for providing timely and comprehensive care to neonates with this genetic disorder.

## Case presentation

**Figure 1:**
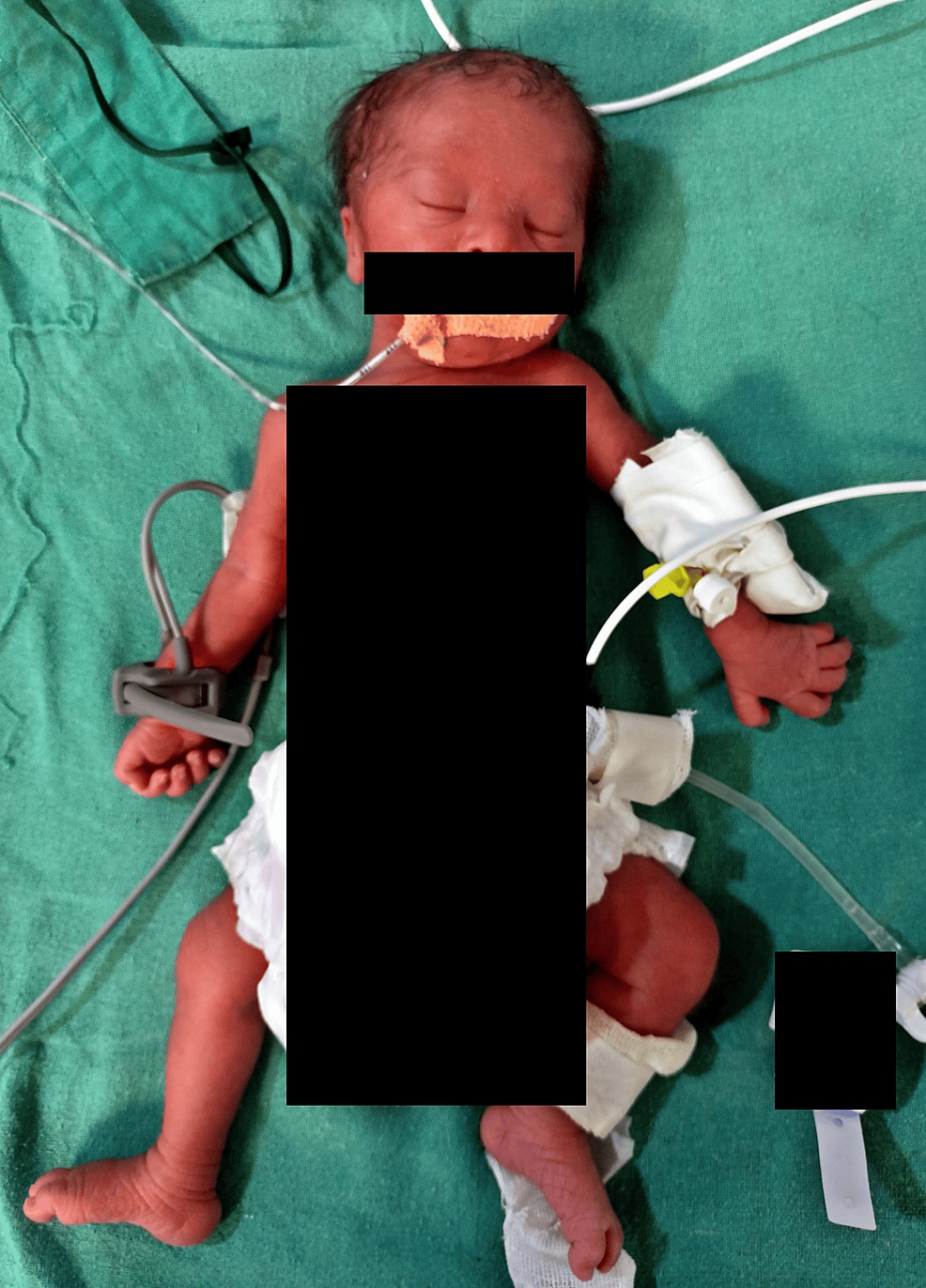
Image shows a depressed nasal bridge, small dysplastic ears, upward slanting of the eyes, flat face, short neck, and joint hyperflexibility

We present the case of a 2 kg male neonate born via normal vaginal delivery on January 2, 2024, at 10 pm in a tertiary care hospital in central India. The infant, initially unresponsive and not crying at birth, was promptly transferred to the neonatal intensive care unit (NICU) due to respiratory distress. Syndromic features were noted, including a depressed nasal bridge, small dysplastic ears, upward slanting of the eyes, a single transverse palmar crease, flat face, poor moro reflex, hypotonia, short neck, and joint hyperflexibility, leading to a clinical diagnosis of Down syndrome confirmed through karyotyping (Figure 1).

Upon admission, the neonate's vitals were stable, and blood investigations revealed a hemoglobin level of 13, total leukocyte count of 9400, hematocrit of 41, and platelet count of 59000. The infant was started on intravenous fluids, ampicillin, and gentamycin. Nasal prong oxygen supplementation was initiated, and orogastric feeds were introduced after eye congestion prompted the addition of tobramycin eye drops. As the baby accepted full oral feeds, intravenous fluids were discontinued.

**Figure 2:**
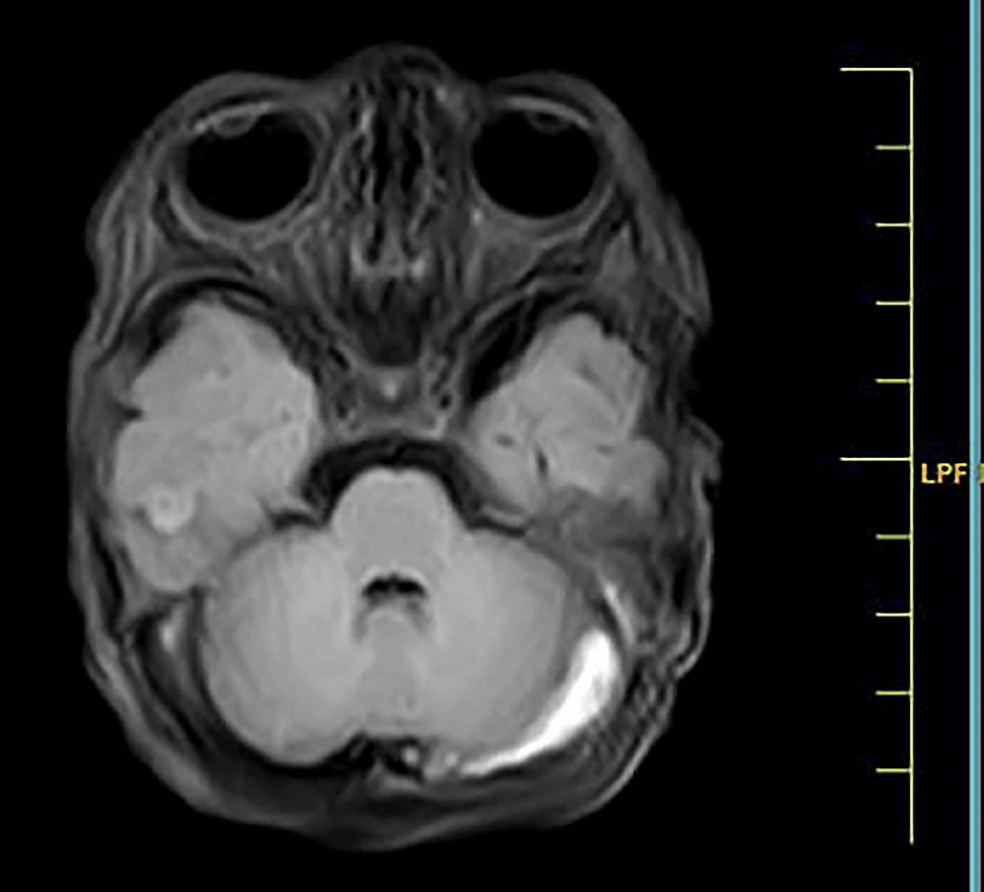
Image shows loss of flow void in the left transverse sinus, suggesting venous sinus thrombosis

The clinical course was complicated by multiple episodes of vomiting, prompting an EEG that indicated moderate to severe hypoxic-ischemic encephalopathy (HIE). A subsequent MRI at day of life 14 revealed the loss of flow void in the left transverse sinus, suggesting venous sinus thrombosis, a rare occurrence in neonates with Down syndrome (Figure 2).

The neonate faced challenges such as aspiration pneumonia, requiring cardiopulmonary resuscitation, intubation, and an upgrade of antibiotics to ceftazidime and vancomycin. A blood transfusion was administered due to decreasing hemoglobin levels. After extubation, the baby exhibited poor respiratory drive, leading to re-intubation. Neurophysiotherapy and oromotor stimulation were initiated to address these challenges. Blood and endotracheal cultures revealed no growth in blood culture and the growth of *Klebsiella pneumoniae* in the endotracheal culture. Antibiotics were adjusted to tigecycline and colistin for seven days, followed by successful discontinuation. The neonate's respiratory status improved, and extubation transitioned to continuous positive airway pressure (CPAP). Upon discharge, the neonate demonstrated improvement in weight (2.230 kg), length (48 cm), and head circumference (33 cm). The parents were educated on breastfeeding and kangaroo care and advised against applying *kajal *(kohl) on the eyes. The case concludes with recommendations for ongoing monitoring, including Brain Evoked Response Auditory (BERA) at three months.

## Discussion

This case report describes the intricate management of a neonate with Down syndrome complicated by dural venous thrombosis, an unusual manifestation in this population. The unique challenges encountered in the course of care highlight the importance of a multidisciplinary approach encompassing neonatology, neurology, and infectious disease management. The presented case aligns with existing literature on Down syndrome, emphasizing the characteristic syndromic features and potential comorbidities associated with this chromosomal abnormality. Previous studies have documented the increased risk of respiratory distress, hypotonia, and congenital heart defects in neonates with Down syndrome [1]. The early identification of these features through karyotyping is crucial for timely intervention and comprehensive care.

Venous thrombosis is a rare but severe complication in neonates, and its association with Down syndrome is scarcely reported. The loss of flow void in the left transverse sinus, as observed in the MRI of our case, has been documented in a few cases, indicating the need for heightened awareness of thrombotic events in neonates with Down syndrome [7]. While the exact mechanism remains unclear, genetic and environmental factors may contribute to this population's increased risk of thrombosis [8]. The complexity of this case necessitated a comprehensive approach involving neonatologists, neurologists, and infectious disease specialists. The prompt recognition of aspiration pneumonia and venous thrombosis, coupled with timely intubation, appropriate antibiotic therapy, and neurophysiotherapy interventions, underscores the importance of a collaborative and adaptive healthcare strategy [9].

The infectious complications in this case, including *Klebsiella pneumoniae* growth, align with previous reports on nosocomial infections in NICUs. The successful resolution following targeted antibiotic therapy highlights the critical role of microbiological surveillance and tailored treatment in managing infections in critically ill neonates [10]. The positive outcome at discharge, with the neonate improving anthropometric measurements and overall clinical status, reinforces the importance of ongoing monitoring and follow-up. Regular assessments, including BERA at three months, are crucial for identifying developmental delays and ensuring appropriate interventions [11].

## Conclusions

In conclusion, the successful management of this intricate case involving a neonate with Down syndrome and dural venous thrombosis underscores the imperative of a multidisciplinary approach in neonatal care. The collaboration among neonatologists, neurologists, and infectious disease specialists played a pivotal role in navigating the challenges, including respiratory distress, aspiration pneumonia, and the rare occurrence of venous thrombosis. The positive outcome at discharge, marked by improved anthropometric measurements and overall clinical status, highlights the effectiveness of timely interventions and vigilant monitoring. This case also prompts further exploration into the mechanisms and risk factors associated with venous thrombosis in neonates with Down syndrome. Overall, it emphasizes the importance of ongoing research to enhance our understanding of such complexities, ultimately contributing to refined clinical practices and improved outcomes for neonates facing intricate medical conditions.
